# Coleta de gordura para injeções de tecido adiposo microfragmentado: Estudo piloto de comparação da segurança de procedimentos realizados por cirurgiões ortopédicos ou plásticos

**DOI:** 10.1055/s-0045-1813004

**Published:** 2025-12-10

**Authors:** Bruno Butturi Varone, Rodrigo Bernstein Conde, Chilan B. G. Leite, Pedro Nogueira Giglio, Riccardo Gomes Gobbi, Marco Kawamura Demange

**Affiliations:** 1 Grupo de Joelho, Instituto de Ortopedia e Traumatologia, Hospital das Clínicas (HCFMUSP), Faculdade de Medicina, Universidade de São Paulo, São Paulo, SP, Brasil; 2 Instituto Universitario de Ciencia de la Salud Fundación H. A. Barceló, Facultad de Medicina, Buenos Aires, Argentina; 3 Departamento de Cirurgia Ortopédica, Brigham and Women's Hospital, Harvard Medical School, Boston, MA, Estados Unidos

**Keywords:** osteoartrite, osteoartrite do joelho, tecido adiposo, adipose tissue, osteoarthritis, osteoarthritis, knee

## Abstract

**Objetivo:**

Este estudo teve como objetivo comparar as taxas de complicações em curto prazo da coleta de tecido adiposo em pequeno volume para injeções de tecido adiposo microfragmentado (mFAT) no joelho realizada por um cirurgião ortopédico ou um cirurgião plástico, além de avaliar a curva de aprendizado de cirurgiões ortopédicos.

**Métodos:**

Este estudo de caso-controle incluiu pacientes com osteoartrite de joelho. Todos os pacientes foram submetidos a um procedimento em estágio único composto por coleta de tecido adiposo abdominal, processamento do material extraído com o dispositivo Lipogems (Lipogems International SpA) para obtenção de mFAT, que foi injetada via intra-articular no joelho. Os pacientes foram divididos em grupo teste, no qual a coleta foi realizada por um cirurgião ortopédico recém-treinado, e grupo controle, no qual o procedimento foi feito por um cirurgião plástico com experiência. Efeitos adversos em curto prazo e complicações mais e menos sérias relacionadas à coleta foram avaliados no período intraoperatório e à consulta de acompanhamento 7 dias após a cirurgia.

**Resultados:**

Nenhum paciente apresentou complicações mais sérias (embolia gordurosa, eventos tromboembólicos, perfuração abdominal, infecção da ferida, deiscência ou alterações estéticas). A taxa de desconforto abdominal durante a coleta, classificado como complicação menor, não apresentou diferença estatisticamente significativa entre os grupos ( *p* = 0,362). Os efeitos adversos pós-operatórios, como equimoses ( *p* = 0,362) e desconforto ( *p* = 0,342) abdominais, foram equivalentes em ambos os grupos e se resolveram em 7 dias.

**Conclusão:**

Este estudo piloto sugere que, com treinamento adequado, cirurgiões ortopédicos podem realizar a coleta de tecido adiposo em pequeno volume com baixas taxas de complicações, comparáveis às observadas em procedimentos realizados por cirurgiões plásticos experientes em lipoaspiração.

## Abstract (en)

**Objective:**

To compare short-term complication rates of small-volume adipose tissue harvesting for micro-fragmented adipose tissue (mFAT) knee injections performed by orthopedic surgeons with those performed by plastic surgeons. Additionally, to evaluate the orthopedic surgeon's learning curve.

**Methods:**

The present case-control study enrolled patients with knee osteoarthritis. All patients underwent a single-stage procedure consisting of abdominal adipose tissue harvesting, processing of the extracted material using the Lipogems (Lipogems International SpA) device to obtain mFAT, which was then injected intra-articularly into the knee. The patients were divided into a test group, with harvesting being performed by a recently trained orthopedic surgeon, and a control group, in which the procedure was performed by an experienced plastic surgeon. Short-term adverse effects, minor and major complications related to harvesting, were assessed intraoperatively and at 7-day follow-up.

**Results:**

No major complications (fat embolism, thromboembolic events, abdominal perforation, wound infection, dehiscence, or cosmetic changes) were observed in either group. Abdominal discomfort during harvesting, classified as a minor complication, showed no statistically significant difference between groups ( *p* = 0.362). Postoperative adverse effects, such as abdominal ecchymosis ( *p* = 0.362) and discomfort ( *p* = 0.342), were equivalent in both groups and resolved within 7 days.

**Conclusion:**

The present pilot study suggests that, with adequate training, orthopedic surgeons can perform small-volume adipose tissue harvesting with low complication rates comparable to those achieved by plastic surgeons experienced in liposuction.

## Introdução

A osteoartrite do joelho (OAJ) é uma doença de alta prevalência que acomete uma parcela significativa da população global. A prevalência geral de OAJ sintomática é estimada em 30%, chegando a 44% em homens e 28% em mulheres com idades entre 55 e 64 anos. 1 Os tratamentos variam de acordo com a gravidade dos sintomas e as necessidades dos pacientes. Terapias injetáveis são amplamente utilizadas para proporcionar alívio da dor em médio prazo. Entre as opções injetáveis, o tecido adiposo microfragmentado (mFAT) ganhou atenção por seus resultados promissores na redução da dor. 2 3 Diversos estudos, inclusive ensaios clínicos, relataram desfechos superiores em pacientes com OAJ tratados com mFAT em comparação àqueles submetidos ao tratamento conservador ou injeções de ácido hialurônico. 4 5 6 7 8

Em estética e reconstrução, a lipoaspiração é considerada um procedimento menor quando o volume extraído é inferior a 1.000 mL. De modo geral, é realizada sob anestesia local, estando associada a baixas taxas de complicações. Por outro lado, lipoaspirações de grande volume (ou seja, > 10% do peso corporal) tendem a exigir anestesia geral e estão relacionadas a riscos maiores, incluindo infecção, hematoma, equimose e embolia gordurosa ou pulmonar. 9

No entanto, na prática ortopédica, o volume de tecido adiposo necessário é mínimo. Estudos sugeriram que a coleta de menos de 50 mL é suficiente para produzir 5 a 10 mL de mFAT, um volume adequado para injeções em articulações maiores, como o joelho. 10 11 Dado o pequeno volume e a natureza minimamente invasiva do procedimento, a anestesia local é, com frequência, suficiente, apoiando a ideia de que, com treinamento adequado, os cirurgiões ortopédicos podem realizar a coleta com segurança.

Embora a lipoaspiração de pequeno volume por cirurgiões ortopédicos seja viável, avaliar sua segurança quando realizada por profissionais não especializados em cirurgia plástica continua sendo essencial. Portanto, este estudo teve como objetivo comparar as taxas de complicações em curto prazo da coleta de tecido adiposo em pequeno volume por cirurgiões ortopédicos ou plásticos para injeções de mFAT em pacientes com OAJ. Nossa hipótese era que as taxas de complicações seriam comparáveis entre as duas especialidades.

## Métodos

Este estudo de caso-controle foi aprovado pelo comitê de ética local (CAAE 52440821.5.0000.0068) e todos os participantes assinaram o termo de consentimento livre e esclarecido. Avaliamos os efeitos adversos e as complicações relacionadas à coleta de tecido adiposo abdominal em pacientes com OAJ. No grupo teste, o procedimento foi realizado por um cirurgião ortopédico recém-treinado e, no grupo controle, por um cirurgião plástico. Todos os pacientes foram submetidos a um procedimento em estágio único, composto por coleta de tecido adiposo abdominal, processamento com o dispositivo descartável Lipogems (Lipogems International SpA) para obtenção de mFAT, seguido de injeção intra-articular de mFAT no joelho acometido.

A elegibilidade dos pacientes para o tratamento com injeções intra-articulares unilaterais ou bilaterais no joelho foi baseada nos sintomas clínicos. Os critérios de inclusão foram pacientes com OAJ sintomática confirmada por avaliação radiográfica, com aceitação de todos os graus de Kellgren-Lawrence (1–4). Os critérios de exclusão foram a presença de deformidade em varo ou valgo acima de 10° e índice de massa corporal (IMC) superior a 40 kg/m ^2^ .

No total, 33 procedimentos foram realizados. Nos primeiros 13 casos, um cirurgião plástico com vasta experiência em lipoaspiração fez a coleta de tecido adiposo enquanto era observado e dava treinamento prático a um cirurgião ortopédico. Os 20 procedimentos restantes foram realizados de forma independente pelo cirurgião ortopédico. Todas as injeções intra-articulares no joelho ocorreram sob orientação ultrassonográfica.

### Coleta de Tecido Adiposo e Processamento de mFAT

**Fig. 1:**
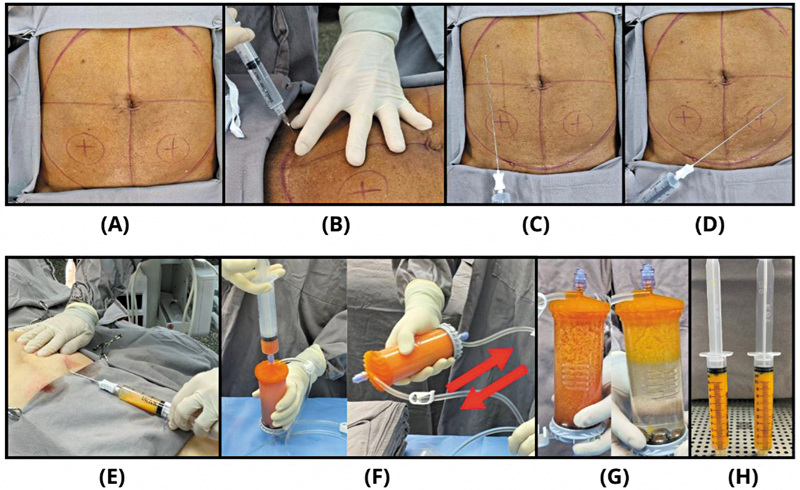
( **A** ) Marcação dos portais cutâneos bilaterais acima da linha inguinal. ( **B** ) Cada portal recebeu 1 mL de lidocaína a 2%. ( **C, D** ) A solução anestésica foi infiltrada em cada hemiabdome. ( **E** ) O tecido adiposo foi coletado usando uma cânula 13G conectada a uma seringa VacLock. ( **F** ) Microfragmentação mecânica do tecido adiposo coletado. ( **G** ) Mudança na aparência do tecido após lavagens e fragmentação sucessivas. ( **H** ) Tecido adiposo microfragmentado (mFAT) pronto para injeção intra-articular no joelho.

A coleta de tecido adiposo abdominal, o processamento com o dispositivo Lipogems e as injeções guiadas por ultrassom foram realizados em um procedimento em estágio único. Os pacientes foram posicionados em decúbito dorsal, em condições estéreis, no centro cirúrgico e submetidos à anestesia local. O abdome inferior foi escolhido como sítio padrão para a coleta devido à acessibilidade e volume consistente de tecido adiposo. Os portais cutâneos foram marcados bilateralmente acima da linha inguinal ( Fig. 1A ) e cada portal recebeu 1 mL de lidocaína a 2% ( Fig. 1B ). Após aguardar o início do efeito anestésico, pequenas incisões, de aproximadamente 4 mm, foram feitas com um bisturi de lâmina número 11.

O tecido adiposo foi coletado por meio de técnica tumescente em dois estágios. 12 A infiltração anestésica foi realizada por meio da introdução de uma cânula em cada portal para distribuição da solução por toda a área de coleta subcutânea. A infiltração utilizou a cânula 19G que faz parte do kit Lipogems. A solução anestésica injetada foi composta por 20 mL de lidocaína a 2%, 20 mL de bupivacaína a 0,5%, 1 mL de adrenalina a 1 mg/mL e 250 mL de solução salina a 0,9%, com volume total de 291 mL. Esse total foi dividido em 120 mL em cada hemiabdome, sendo os 51 mL restantes reservados para anestesia adicional, caso necessária ( Figs. 1C e D ). Depois do período de latência apropriado, o tecido adiposo foi coletado com a cânula 13G (também parte do kit Lipogems) conectada a uma seringa VacLock (Merit Medical Systems Inc.), como apresentado na Fig. 1E . A coleta foi feita de maneira uniforme, evitando extrações repetidas perto dos portais para minimizar possíveis problemas estéticos.

A gordura subcutânea remanescente foi avaliada por meio de pinçamento repetido da área, uma técnica confiável para assegurar a permanência de uma camada adequada de tecido e evitar alterações estéticas. 13 Nos pacientes com OAJ unilateral, foram coletados 60 mL de tecido adiposo, enquanto aqueles com bilateral foram submetidos à coleta de 120 mL. Esses volumes foram padronizados com base em estudos anteriores que indicam a quantidade de tecido adiposo necessária para produção de 10 mL de mFAT por joelho. 14

Após a coleta, os portais cutâneos foram fechados com suturas de nylon 5.0. O tecido adiposo foi processado com o dispositivo descartável Lipogems, seguindo protocolos estabelecidos por estudos anteriores ( Figs. 1F–H ). 2 O mFAT resultante foi injetado no joelho por via superolateral, sob orientação ultrassonográfica, com agulha 16G. Não houve necessidade de imobilização e os pacientes foram incentivados a iniciar exercícios de amplitude de movimento do joelho no primeiro dia após o procedimento.

### Coleta de Dados

Dados demográficos, incluindo idade, sexo e IMC, foram coletados de cada paciente. O volume de tecido adiposo coletado e a quantidade de mFAT obtido também foram registrados. As complicações relacionadas à coleta foram avaliadas no mesmo dia e novamente em uma consulta de acompanhamento após 7 dias. As complicações foram categorizadas de acordo com o sistema de Clavien-Dindo, da seguinte forma:

- As complicações mais sérias (grau de Clavien-Dindo III ou superior) incluíram embolia gordurosa, eventos tromboembólicos venosos, perfuração abdominal, infecção de ferida com necessidade de intervenção cirúrgica, infecção intra-articular e alterações cosméticas significativas.
- As complicações menos sérias (graus de Clavien-Dindo I e II) incluíram eventos adversos brandos ou esperados, como equimoses abdominais, desconforto ou outras complicações que não provocaram comprometimento residual.

### Análise Estatística

As variáveis categóricas foram relatadas como o número de eventos e as porcentagens correspondentes, enquanto as variáveis contínuas foram apresentadas como média ± desvio-padrão (DP). O teste de Shapiro-Wilk foi utilizado para avaliar a normalidade das variáveis contínuas. Dependendo da distribuição, as comparações entre as variáveis contínuas foram feitas com o teste *t* ou de Mann-Whitney. O teste exato de Fisher foi utilizado para comparação das variáveis categóricas. A significância estatística foi definida como *p* < 0,05.

## Resultados

O estudo incluiu 33 pacientes. Desses, 13 foram submetidos à coleta de tecido adiposo por um cirurgião plástico e 20 por um cirurgião ortopédico. A média de idade foi de 61,2 ± 10,2 anos no grupo do cirurgião plástico e 60,4 ± 8,6 anos no grupo do cirurgião ortopédico ( *p* = 0,802). O IMC médio foi de 27,7 ± 4,5 kg/m ^2^ no grupo do cirurgião plástico e 29,3 ± 4,6 kg/m ^2^ no do ortopédico ( *p* = 0,349).

O volume médio de tecido adiposo coletado não diferiu significativamente entre os grupos (plástico: 117,7 ± 15,4 mL; ortopédico: 123,0 ± 33,8 mL; *p* = 0,245). Da mesma forma, o volume de mFAT obtido após o processamento do tecido adiposo foi comparável entre os grupos (28,5 ± 3,1 mL e 27,4 ± 11,1 mL; *p* = 0,645).

Nenhum paciente apresentou complicações mais sérias. Houve duas complicações menos sérias, descritas como desconforto do paciente durante o procedimento de coleta, foram relatadas em cada grupo. À consulta de acompanhamento 7 dias após a cirurgia, equimoses abdominais foram observadas em 12 pacientes (92,3%) no grupo do cirurgião plástico e em 17 (85,0%) no do ortopédico. O desconforto abdominal foi relatado por 11 pacientes (84,6%) no grupo do cirurgião plástico e 16 (80,0%) no ortopédico. As características basais dos pacientes e os desfechos do procedimento estão resumidos na Tabela 1 .

**Tabela 1:** Características dos pacientes e eventos adversos de acordo com a especialidade do cirurgião

	Cirurgião ortopédico	Cirurgião plástico	Valor de *p*
Número de casos	20	13	
Idade (anos)	60,4 ± 8,6	61,2 ± 10,2	0,802
Sexo (feminino/masculino)	17/3	10/3	0,659
IMC (kg/m ^2^ )	29,3 ± 4,6	27,7 ± 4,5	0,349
OAJ bilateral	12 (60)	11 (84,6)	0,246
Volume de tecido adiposo (mL)	123,0 ± 33,8	117,7 ± 15,4	0,245
Volume de mFAT (mL)	27,4 ± 11,1	28,5 ± 3,1	0,645
Complicações menos sérias	2 (18,2)	2 (10)	0,362
Equimose abdominal	17	12	0,362
Desconforto abdominal	16	11	0,342

**Abreviações:** IMC, índice de massa corporal; OAJ, osteoartrite do joelho; mFAT, tecido adiposo microfragmentado.

**Nota:** Valores expressos como média ± desvio padrão (DP) ou número (porcentagem).

## Discussão

O principal achado deste estudo piloto é que, com treinamento adequado, cirurgiões ortopédicos podem realizar a coleta de tecido adiposo para injeções de mFAT com segurança e eficácia. As taxas de complicações foram igualmente baixas em procedimentos feitos por cirurgiões ortopédicos ou plásticos. Além disso, os volumes semelhantes de tecido adiposo coletados e processados pelos dois grupos corroboram a viabilidade de realização deste procedimento por cirurgiões ortopédicos.

Notavelmente, o volume médio de mFAT obtido por paciente foi quase três vezes maior do que a quantidade usual necessária para uma única injeção em uma articulação maior, como o joelho. Isso tem particular relevância em pacientes submetidos a injeções unilaterais, em que volumes menores podem ser suficientes.

Estudos anteriores relataram baixas taxas de complicações em lipoaspirações, mesmo de volumes maiores. Por exemplo, Aljerian et al. 15 conduziram uma meta-análise incluindo 60 estudos e 21.776 pacientes, relatando uma taxa geral de complicações de 12%, em especial equimoses e edema. Com a exclusão destes efeitos colaterais, a taxa de complicações menores caiu para 5%, sendo observadas complicações maiores em apenas 1% dos casos. As complicações menores incluíram alterações cosméticas, seroma, hematoma, equimoses e edema, enquanto as complicações maiores abrangeram trombose venosa profunda, sepse, lesão visceral, hipovolemia e distúrbios pulmonares. É importante notar que essa meta-análise se concentrou em procedimentos de lipoaspiração de alto volume para fins estéticos.

Chow et al. 16 investigaram os limites de segurança da lipoaspiração e concluíram que a coleta de mais de 100 mL por unidade de IMC pode aumentar os riscos de complicações. Consequentemente, em um paciente com IMC de 30 kg/m ^2^ , volumes de até 3.000 mL ainda foram considerados seguros. Vale ressaltar que esses procedimentos foram realizados sob anestesia geral por cirurgiões plásticos com certificação.

Outra consideração importante se refere ao possível risco de toxicidade anestésica. A literatura sugere o uso seguro de lidocaína até 35 mg/kg e, apesar da dose relativamente alta usada em técnicas tumescentes, essa abordagem continua sendo aceita como segura por várias razões. 17 Primeiro, embora a técnica exija um grande volume de solução anestésica diluída, a adição de adrenalina retarda a absorção de lidocaína, cujos picos plasmáticos geralmente ocorrem 12 a 14 horas após o início da infiltração. Além disso, a lipoaspiração em si reduz a quantidade total de lidocaína absorvida de maneira sistêmica. A técnica também minimiza a perda sanguínea devido aos efeitos vasoconstritores da adrenalina. Ao ponderar esses fatores, optamos por empregar a técnica tumescente, que se mostrou eficaz em minimizar o desconforto abdominal durante a coleta de tecido adiposo.

Em relação ao volume injetado, em nosso estudo, cada joelho acometido recebeu 10 mL de mFAT. Embora esse volume pareça apropriado para articulações maiores, as menores podem exigir volumes ainda mais baixos. Em uma série de casos de pacientes com OAJ, Dall'Oca et al. 18 relataram a coleta de 60 mL de tecido adiposo, com obtenção de 5 a 10 mL de mFAT; essa quantidade foi associada a uma redução efetiva da dor e à melhora dos desfechos clínicos. Da mesma forma, D'Ambrosi et al. 19 coletaram 45 mL de tecido adiposo e injetaram 5 mL de mFAT em tornozelos artríticos, também com desfechos clínicos favoráveis. Esses exemplos destacam que, de modo geral, as aplicações ortopédicas requerem volumes menores em comparação a procedimentos estéticos, mesmo para procedimentos bilaterais em articulações maiores. Essa distinção é importante, pois corrobora a viabilidade da realização de tais procedimentos em ambulatórios, o que é bastante relevante dada a alta prevalência da osteoartrite e a importância da contenção de custos. Portanto, ao diminuir a hospitalização, o tempo no centro cirúrgico e os custos de anestesia, essa abordagem pode ampliar o acesso a tratamentos regenerativos.

Em nosso estudo, o desconforto do paciente durante o procedimento, incluindo relacionado a posicionamento inadequado ou à coleta, foi classificado como uma complicação menor. Como todos os procedimentos ocorreram sob anestesia local, esses eventos foram mais frequentes durante o início da curva de aprendizado. No entanto, à medida que os cirurgiões ortopédicos e plásticos ganharam experiência, tais ocorrências se tornaram menos comuns. Atribuímos esse desconforto inicial ao menor ritmo do procedimento nos primeiros casos. Isso destacou a importância do posicionamento ideal e ergonômico do paciente, em especial sob anestesia local. Adicionalmente, o mapeamento cuidadoso da área de coleta e a infiltração completa com a solução tumescente pareceram importantes para minimizar o desconforto durante a coleta. Embora tenhamos preparado 291 mL da solução de Klein para anestesia local, apenas 240 mL foram administrados (120 mL por hemiabdome). O volume restante foi reservado para uso em caso de necessidade de anestesia adicional.

Nenhuma complicação maior foi observada em nosso estudo piloto. Outros estudos que avaliaram a segurança do mFAT para OAJ também relataram taxas semelhantes e baixas de complicações relacionadas à coleta de tecido adiposo. 20 21 22 23 Da mesma forma, Aljerian et al. estimaram baixas taxas de complicações maiores (1%), mesmo em procedimentos de lipoaspiração de grande volume. 15

Lesões viscerais e vasculares causadas por agulhas de infiltração ou cânulas de lipoaspiração são raras e foram relatadas principalmente em pacientes com cicatrizes ou hérnias abdominais ou ainda em casos de alto volume sob anestesia geral. 24 25 26 Em nosso estudo, todos os pacientes foram submetidos a uma avaliação pré-operatória completa e as incisões abdominais anteriores foram evitadas por meio do emprego da técnica de dois portais. Além disso, o desconforto abdominal e as equimoses foram previstos como efeitos colaterais comuns. Esses eventos foram discutidos com todos os pacientes durante o processo de consentimento e medicamentos tópicos ou analgésicos foram usados para atenuá-los.

Nosso estudo não está isento de limitações. O cirurgião plástico realizou os 13 primeiros procedimentos, enquanto os 20 procedimentos subsequentes foram feitos pelo cirurgião ortopédico. Assim, é plausível que a equipe cirúrgica tenha se familiarizado com o protocolo ao longo do tempo, o que pode ter melhorado a eficiência nos casos posteriores. No entanto, é importante notar que o cirurgião plástico já tinha experiência prévia com lipoaspiração em outros contextos clínicos. Por outro lado, o cirurgião ortopédico não possuía experiência prática prévia e teve apenas treinamento observacional limitado durante os primeiros casos, tornando improvável que essa breve exposição superasse a expertise do cirurgião plástico.

Outra limitação é a ausência de um cálculo formal do tamanho da amostra, o que, embora aceitável para um estudo piloto, limita a generalização dos achados. Portanto, estudos clínicos randomizados com delineamentos específicos para comparação dos resultados da coleta de tecido adiposo por diferentes especialidades cirúrgicas poderiam fornecer evidências mais robustas. No entanto, até onde sabemos, este é o primeiro estudo a comparar as taxas de complicações entre cirurgiões ortopédicos e plásticos. Nossos achados contribuem para a literatura ao mostrar que a coleta de tecido adiposo em pequeno volume pode ser realizada com segurança por cirurgiões ortopédicos treinados.

## Conclusão

Avaliar a segurança da coleta de tecido adiposo em pequeno volume realizada por cirurgiões ortopédicos é essencial para ampliar o acesso a esse tratamento. Nossos achados corroboram a ideia de que, com treinamento adequado, cirurgiões ortopédicos podem alcançar baixas taxas de complicações ao coletar volumes limitados de tecido adiposo para aplicações ortopédicas. Como um estudo exploratório, esses resultados fornecem uma base para pesquisas futuras que visam analisar a curva de aprendizado e comparar os resultados em diferentes sítios de coleta.
